# Young Children Understand the Normative Implications of Future-Directed Speech Acts

**DOI:** 10.1371/journal.pone.0086958

**Published:** 2014-01-29

**Authors:** Karoline Lohse, Maria Gräfenhain, Tanya Behne, Hannes Rakoczy

**Affiliations:** 1 Institute of Psychology & Courant Research Centre, “Evolution of Social Behaviour”, University of Göttingen, Göttingen, Germany; 2 Department of Child and Adolescent Psychiatry, Psychotherapy and Psychosomatics, University of Leipzig, Leipzig, Germany; Durham University, United Kingdom

## Abstract

Much recent research has shown that the capacity for mental time travel and temporal reasoning emerges during the preschool years. Nothing is known so far, however, about young children's grasp of the normative dimension of future-directed thought and speech. The present study is the first to show that children from age 4 understand the normative outreach of such future-directed speech acts: subjects at time 1 witnessed a speaker make future-directed speech acts about/towards an actor A, either in imperative mode (“A, do X!”) or as a prediction (“the actor A will do X”). When at time 2 the actor A performed an action that did not match the content of the speech act at time 1, children identified the speaker as the source of a mistake in the prediction case, and the actor as the source of the mistake in the imperative case and leveled criticism accordingly. These findings add to our knowledge about the emergence and development of temporal cognition in revealing an early sensitivity to the normative aspects of future-orientation.

## Introduction

Time is a fundamental category that structures basically all of our thinking. From the point of view of cognitive development, the fundamental question is how children's understanding of time emerges and develops with age. Recent research has begun to investigate the ontogeny of temporal cognition systematically and comprehensively. Inspired by work in comparative psychology, much of this research focuses on our ability to mentally travel back and forth in time, so to speak, to remember the past and to imagine the future – often referred to as “mental time travel” [1]–[3]. A related line of work has investigated the development of the capacity to engage in tensed thought: to understand that any event happened, happens or will happen at a particular point in time that has particular relations to the present and to other events in time [4]–[8].

Empirically, these lines of research have shown that both the capacity to remember and reason about past events and the capacity to foresee and imagine future ones emerge in the course of the preschool years. First, regarding the past, children around age four start to identify with their past selves in the present and show delayed self-recognition [9], [10], and they use temporal language such as “yesterday” in their spontaneous speech [11], [12]. From this age on, children also begin to reason systematically about the temporal-causal relations of events, e.g. when inferring consequences from the order of events that they experienced in the past [13]–[16].

Second, regarding the future, 4-year olds are able to inhibit a present desire in favor of a future reward [17]. They are likely to succeed in saving and planning tasks [18], [19], and they correctly respond to temporal terms such as “tomorrow” in future-oriented communication [11].

Third, the two capacities emerge in synchrony and correlated fashion between age 3 and 5. Joint emergence and systematic correlations between past and future cognition have been found, for example, in language understanding (“yesterday”/“tomorrow”) [20] and tasks involving the concept of a past self (delayed self-recognition) and the concept of a future self (delay of gratification) [17]. In addition, adult neuroscientific work suggests shared underlying neural substrates for episodic memory and episodic foresight [21], [22]. Taken together, these findings suggest a common underlying cognitive basis for thinking about past and future [20], [23].

So far, however, one central aspect of tensed thinking has received basically no attention in this kind of research: namely the *normative dimension* of our temporal thought and speech. Our mental states and speech acts reach out into the past and into the future. When they do so, in particular when reaching out into the future, they can do this -even with the same propositional content- in two fundamentally different ways: (i) representing the future as it (subjectively) will be, or (ii) representing the future as it (subjectively) ought to be from one's point of view. Paradigmatic mental states of type (i) are beliefs about the future, and the paradigmatic corresponding speech acts are assertions about the future (predictions), such as “Peter will eat the cake”. These have the so-called *mind-to-world* direction of fit [24], [25], aiming at representing the world truly and accurately. If the propositional content of “Peter will eat the cake” is not fulfilled, the mistake is on the part of the speaker. Paradigmatic mental states of type (ii) are desires about future events, typically expressed in imperative speech acts like “Peter, eat the cake!” These have the so-called *world-to-mind* direction of fit, aiming at bringing the world in line with the content of the mental state/speech act. When the propositional content of “Peter, eat the cake!”, – which is in fact the very same propositional content as in the case of the prediction “Peter will eat the cake” - namely the proposition <that Peter will eat the cake> is not fulfilled, the mistake is now not on the part of the speaker, but on the part of the addressee.

Different kinds of speech acts such as assertions and imperative speech acts can take the very same content (e.g. the proposition <that Peter will eat the cake>) but differ in their mode – much like different kinds of propositional attitudes such as believing and desiring can have the same content (e.g. <that Peter will eat the cake>) while differing in psychological mode. Now, what determines the mode of a propositional attitude or a speech act? In the case of propositional attitudes, the mode is essentially constituted by the functional role of a given type of attitude – by what job this attitude does in the mental economy of the subject (e.g. beliefs are attitudes that aim at tracking reality and are therefore sensitive to perceptual evidence, that lead inferentially to other beliefs, and that together with desires rationalize and lead to actions [26]–[28]. The mode of a speech act, in contrast, is largely, but not exclusively determined by the psychological attitude of the speaker. For example, although imperative speech acts are largely constituted by the expression of a desire to someone else, not any utterance that expresses a desire towards someone constitutes an imperative speech act. For each given type of speech act, there are specific background conditions, varying from one type of speech act to another, that have to be met in order for such a speech act to successfully materialize [24]. More specifically, imperative speech acts have some such success conditions that do not apply to other speech acts, assertions in particular. These conditions include the following: the imperative is reasonable, the speaker is in a position to reasonably ask the addressee to perform the action (for example, if I step up to a stranger and say” Give me the moon!”, this fails to constitute a successful imperative speech act…), and the addressee acknowledges the imperative (e.g. “Okay!”). Only if these conditions are met, has the speaker performed a successful imperative and has an obligation been transferred on the addressee.

If such conditions are met, due to their different logical structures and normative forces, future-directed assertive and imperative speech acts engender very different normative relations to the future in speakers and addressees: speakers of assertions are committed to the truth of predicted future states of affairs whereas addressees of imperatives are committed to bringing about the desired states of affairs.

From the point of view of cognitive development, the fundamental question is how children's grasp of these different kinds of *cognitively reaching out into the future* emerges and develops. Existing studies on pragmatic development suggest that children's understanding of the logical structure of future-directed speech acts develops rather late, between the ages of 7 and 9 [29]–[31]. This research shows that children around 5 to 6 years of age find it difficult to distinguish the different kinds of commitments engendered by predictions and promises (note that promises are basically imperatives to oneself [24]): When asked whether someone promised or predicted something, children judged any speech acts - predictions and promises alike - as predictions when they were unfulfilled, and as promises when their content came true. Only beginning with age 9 did children discriminate predictions and promises by holding speakers *responsible* for the fulfillment when the speech act was a promise, but not if the speech act was a prediction.

What these results might suggest is that it is not before well into school age that children come to differentiate the underlying normative force and directions-of-fit of different types of future-oriented speech acts. However, such a strong conclusion clearly might not be warranted by the data. First, the tasks used so far are quite demanding, as children had to follow, memorize and to judge hypothetical stories instead of perceiving the critical events directly. Second, participants had to judge the stories they were presented with by verbally responding to a series of experimenter-questions, which again draws on the presence of sophisticated memory, and particularly, on language skills. It is thus possible that the methodology of previous studies might have seriously underestimated young children's competence and produced false negatives.

In fact, recent research investigating children's understanding of speech-acts with alternative methods might be compatible with this hypothesis; For *present-tense* speech acts, it has been shown that children as young as two to three years of age are able to differentiate the *direction of fit* of speech-acts with the same propositional content. They selectively criticized a speaker for a false assertion of the type “Actor does X” (to the effect that the actor was doing Y at the time of the utterance), but the actor for not complying with a speaker's imperative (“Actor, do X!” with the actor performing a different action at the time of the utterance) [32].

The rationale of the present study was therefore to investigate children's understanding of the normative dimension of *future-oriented* thought and language with a similar methodology. In particular, we tested whether young children understand the normative commitments of different types of future-directed speech acts (predictions vs. imperatives) characterized by different directions of fit, as indicated in their differential protest in the case of mismatches (criticizing the speaker more often than the actor after unfulfilled predictions, but showing the reverse pattern after unfulfilled imperatives).

## Study 1

### Method

Sixteen 4-year-olds, (48–58 months, mean age  = 53 months; 8 boys) were tested (one additional child was excluded due to experimental error). Children were native German speakers, came from diverse socioeconomic backgrounds and were tested either in their daycare centers or in the child lab facilities. Parents gave their written consent for the participation of the children.

This research was conducted in accordance with the Declaration of Helsinki and the Ethical Principles of the German Psychological Society (DGPs), the Association of German Professional Psychologists (BDP), and the American Psychological Association (APA). It involved no invasive or otherwise ethically problematic techniques and no deception (the only cases in which a separate vote by a local ethics committee might be required; see the regulations on freedom of research in the German Constitution, §5 (3), and the German University Law, §22).

### Design & Procedure

In a *warm-up phase* Children were introduced to two hand puppets (a sheep and a hedgehog) that were located in separate rooms of a large toy house, both facing the child. The puppets were operated by a second experimenter (E2) sitting behind the toy house. After a short familiarization phase the first experimenter (E1) presented two warm-up games which were played by the child and the puppets taking turns. First, E1 asked one after another to label objects depicted in a picture book. In the second game a small hammer was used to push one of three differently colored balls through a hole. In the course of these games both puppets repeatedly made mistakes by mislabeling objects and by hitting balls of the wrong color. The aim of the warm up phase was to establish the fact that the puppets might need the child's help. Therefore children were asked to take care that the puppets' actions (verbal and physical) were all correct. In case of a child not correcting mistakes spontaneously, E1 encouraged her to help the puppets play the game the right way.

In the *test game*, in a within-subjects design each child participated in two kinds of trials administered in two blocks of 4 trials each (order counterbalanced). Both kinds of test trials followed the same structure and consisted of the same sliding game: one of the puppets (the speaker) uttered a speech-act referring to the other puppet's (the actor's) future action (sliding an object (e.g. a bird) into its corresponding container (e.g. a nest) – with the child placing the corresponding container at the end of the slide). As the actor's object choice was invisible to the child, the child relied on the content of the speech act in order to choose which container would match the object that the actor would later send down the slide. In all test trials the propositional content of the speech act was never fulfilled by the action. This became obvious by the mismatch of object (e.g. fish) and container (e.g. nest) after the action. The crucial difference between the two test blocks was the type of speech act: in the *prediction (future-assertive)* condition, the speaker's prediction about the actor's action did not come true, e.g. “I guess/I think the hedgehog will slide the bird.” (“Ich glaube, der Igel wird den Vogel rutschen lassen” – where the German “Ich glaube” translates with “I guess” or “I think”), with the actor sliding the fish later on, whereas in the *imperative (future-directive)* condition the speaker's imperative was not fulfilled by the actor, e.g. “Hedgehog, slide the bird next!” (“Igel, lass gleich mal den Vogel rutschen!”), again with the actor sliding the fish afterwards (see Table S1 for a detailed script of the two conditions).

It might look like the propositional content of the assertion “I guess/I think the hedgehog will slide the bird.” is actually not about the hedgehog and what it will do, but rather about the speaker and her belief what the hedgehog will do and therefore has quite a different propositional content from the imperative which clearly is about what the hedgehog will (ought to) do. But the appearances are misleading here. The standard use of “I guess” and “I think” is not to report a belief but to express it ([33], see [34] for developmental data), more specifically to qualify the belief as not utterly certain (one wouldn't say “I guess/I think 1+1 = 2”). Mostly, “I guess”/“I think” function as a qualifier expressing some degree of uncertainty, much like “probably”, and this is also the way it was used here. There were two specific reasons for adding such a qualification to the expression of the speaker's prediction: first, to make the speech act more natural. The speaker made a prediction without good evidence, in which case a prediction without such a qualification would have sounded strange. The second reason was to avoid possible mis-readings of the prediction as indirect imperative. The underlying problem here is that the surface form of predictions “the actor will do X” can be and often are used to make indirect imperatives (“all students will do their coursework until next week”, “you will clean up your room”).

**Figure 1:**
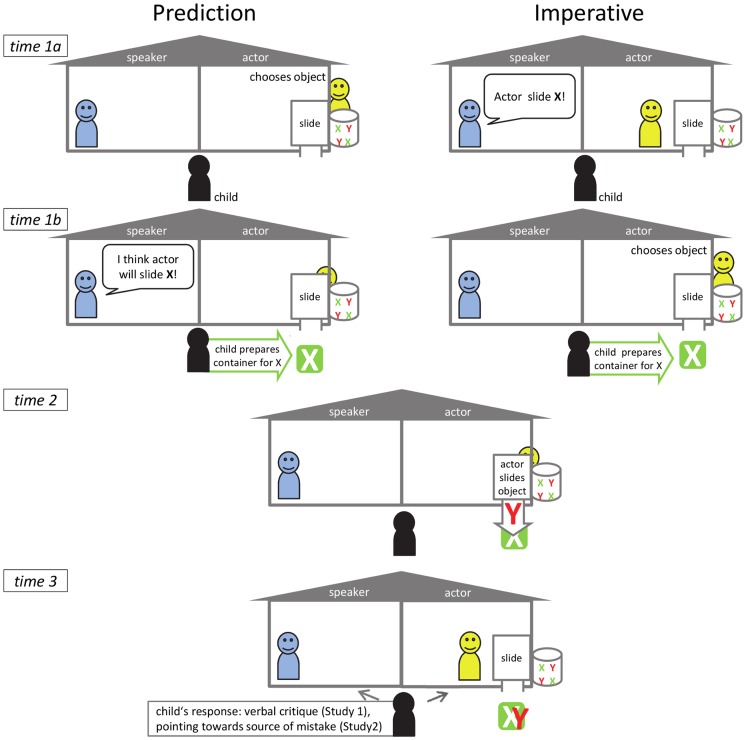
Schematic overview of the procedure. chematic overview of the procedure in imperative and the prediction conditions (Study 1 and Study 2).

The test *procedure* was structured as follows: before each test block, E1 introduced the object-container pairs to the child, asking her to help and find the correct match for each object (for details regarding the material, see Materials S1 and Materials S2). After the child had played with the slide, the objects and the containers herself, the game was given to the puppets. Only the containers remained with the child. At the beginning of each *prediction (future-assertive) trial* the actor puppet disappeared behind the slide in order to choose an object which he placed at the opening of the slide. Then, while the actor was still absent, the speaker puppet told the child which object he thought the actor might play with (see Fig. 1). For the *imperative (future-directive) trials* the speaker puppet declared which object the actor should play, the actor agreed (see Introduction for the necessity of agreement on an imperative for it being valid) and then disappeared behind the slide in order to select the object. After the child had prepared the slide's end with a container, in both conditions the actor slid an object different from the type that was announced by the speaker (see Fig. 1, time 2). When the object had gone down the slide, the puppets remained visible to the child in their rooms (time 3). Children could first react spontaneously to the situation. Second, E1 asked them to explain what had happened.

The puppets' roles of speaker and actor alternated from trial to trial. In order to prevent children from habituating to mismatches in the course of the session, we included a non-test correct trial in the middle of each block where speech act and action matched. Sets of objects and containers were introduced to the child at the beginning of each test block. The first set consisted of miniature animals that were to be slid into their corresponding housings (e.g. bird-nest, fish-aquarium). In the second block miniatures of common object-container pairs were used (e.g. fried egg-pan, car-garage). The order of conditions, the assignment of games to conditions, and the order of the puppet's roles (speaker vs. actor) within each condition were counterbalanced across all children.

### Coding

All sessions were videotaped, one camera capturing the child's face and another capturing gestures and interactions towards the puppets. The data were coded from tape by a single observer. For all 8 test-trials coding started with the moment where the mismatch between speech act *[object type expected]* and action *[object type played]* was visible to the child, i.e. when the object had gone down the slide. Children's verbal responses towards the puppets, as well as their explanations towards E1 were assigned to the following hierarchical categories:

1. *Speaker- or actor-directed protest*: The child clearly criticized one of the puppets by calling its name and/or referencing to its mistake (e.g. “You said he slides the bird but you were wrong!” in response to the speaker, or “Hedgehog, look, this is not the bird! You did it wrong! ” in response to the actor).
2. The code *ambiguous protest* was assigned in two cases: Either when it was indeterminable for the observer which of the puppets was being criticized (e.g. “No! That's wrong!” without observable direction of gaze and/or gesturing). Or, when the child explicitly criticized both puppets (e.g. to E1 “Oh no, the puppets were wrong again!”)

As the focus was on the most sophisticated forms of protest children produced, each trial received as score the highest score observed in the child's response; e.g. in case of a child first criticizing the actor directly (1) and then simply saying to E1 “It was wrong!” (2), this trial was scored as *actor-directed protest* (1). In the very rare case of a child criticizing in the same trial one puppet first and later on the other, the code for *ambiguous protest* was assigned to that trial, as the child's criticism was directed to both speaker and actor.

A second independent observer blind to the hypothesis of the study coded a random sample of 25% of the sessions for inter-rater reliability which was very good (κ = .79).

### Results

**Figure 2:**
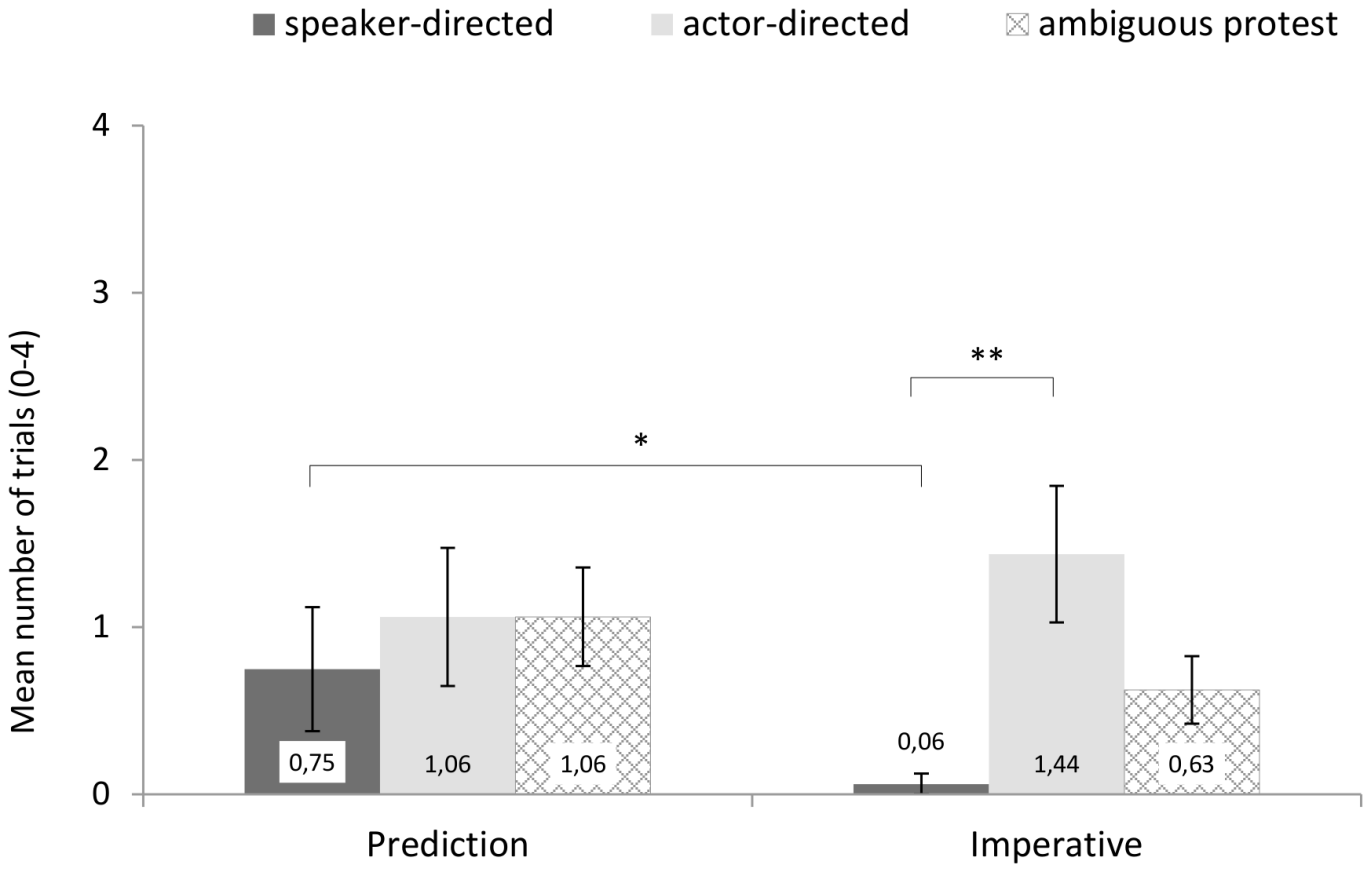
Responses to mismatches in Study 1 (verbal protest measure). ean scores of trials (0–4) per condition (Prediction, Imperative) with each kind of protest (speaker-directed, actor-directed, ambiguous).

For each child, and for the two types of unambiguous protest (speaker-directed and actor-directed) and for ambiguous protest, sum scores across the 4 tasks per condition were computed in which the child showed this kind of protest. The mean sum scores are depicted in Fig. 2. As preliminary analyses found no significant effects for the order of test blocks (mixed factors ANOVAs, n.s.), this factor was not included in the subsequent analyses. As the crucial analyses were based on specific, directed hypotheses (more protest against the speaker than against the actor after unfulfilled predictions, and vice versa after unfulfilled imperatives. And relatedly, more protest against the speaker after unfulfilled predictions than after unfulfilled imperatives, and vice versa for protest against the actor), 1-tailed tests were used. In light of the relatively small sample size, the results of parametric analyses were complemented by non-parametric ones.

First, we compared actor-directed vs. speaker-directed protest within each condition. In the imperative condition, children criticized the actor significantly more often than the speaker, *t*(15)  = 3.22, *p*<.01, *d* = .81. This was confirmed by non-parametric analyses, Wilcoxon test, *T* = 10, *p*<.05, *r* = .65. In the prediction condition, however, there were no significant differences between actor-directed and speaker-directed responses, *t*(15) = .49, *p* = .32 (Wilcoxon test, *T* = 32.5, *p* = .30), with a large proportion of children's responses coded as ambiguous protest (see Fig. 2).

Testing for differences within conditions might neglect performance factors, such as the prepotency of one response type: in both conditions the actor's action directly preceded the apparent mismatch between object and container and so the actor (and her action) was much more salient than the speaker and her previous speech act. Therefore, when deciding who caused the mismatch and who to criticize, especially in case of predictions, children needed to overcome a bias towards the actor, induced by the temporal succession of events (a bias that might have contributed to the high proportion of ambiguous responses in the prediction condition).

Thus, in a second analysis we tested for differences in a given form of directed critique as a function of condition: Do children criticize the speaker more often in the prediction condition than in the imperative condition, and analogously for actor critique? These comparisons showed that children directed critique towards the speaker in prediction trials significantly more often than in imperative trials, *t*(15) = 2.03, *p*<.05, *d* = .51. Again, non-parametric tests confirmed this result, Wilcoxon test, *T* = 44, *p*<.01, *r* = .46. Regarding actor critique, differences between conditions were non-significant, *t*(15) = .79, *p* = .22, (Wilcoxon test, *T* = 29.5, *p* = .20.).

### Discussion

All in all, these findings suggest that children do differentiate between the different kinds of speech acts to some degree, criticizing speaker or actor systematically as a function of condition. These findings were very clear regarding the imperative condition, and regarding the critique of the speaker, but were somewhat less clear regarding the prediction condition, and the critique of the actor.

One fundamental difficulty with the prediction condition compared to the imperative condition might lie in the ambiguity of the linguistic form vis-à-vis different speech act types: in the prediction condition, the linguistic form “The actor will do X” is more ambiguous in that this form can be used to make predictions (the paradigmatic case), but in exceptional circumstances also to utter commands (think of the coach saying to his players “All players will be on the pitch 10 minutes before the game”). The imperative form “Actor, do X!”, in contrast, admits of less ambiguity. Three points should be mentioned in response to this concern: First, given this is a general asymmetry on the level of linguistic form, there is no way around this asymmetry in tests of children's understanding of the two kinds of *direct* future-directed speech acts (it is an interesting question, of course, when children come to understand *indirect* future-directed commands such as “All players will be at the pitch…”. This, however, is a much more complex achievement going well beyond the more fundamental competence under study here). Second, since one can utter an indirect imperative only by talking to an addressee, the ambiguity in the prediction cases arises pragmatically only in situations where the speaker (e.g. the coach) talks to the actor(s) (e.g. the players). Given in our prediction-scenario the actor was not attending to the speech act (the actor puppet left the house and was invisible behind the slide) and the speaker did not explicitly address the actor, this ambiguity does not even seem to apply. Third, and crucially, the structural difference between linguistic forms in imperative and prediction speech acts only poses a problem given the current negative findings in the prediction condition. If one could improve the tasks by removing other potential limiting performance factors, and then document competence after both imperatives and predictions (and for both actor critique and speaker critique), this would show children can track the different directions-of-fit and their normative implications *despite* superficial ambiguities.

Now, one such potential factor in the current study was the dependent measure: A fundamental problem, in particular in the prediction condition, was the high rate of ambiguous responses, i.e. forms of critique that could not be unambiguously assigned to one of the two puppets. Now, these responses *might* reflect children's lacking understanding of the normative structure of predictions. Alternatively, however, the measure might have been too insensitive to uncover children's true competence and thus might have under-estimated children's understanding.

## Study 2

Study 2, thus, followed up on the first study with a modified methodology that aimed to disambiguate children's responses. The same basic scenarios were used, but instead of the verbal protest measure a forced choice paradigm was introduced: children were asked to decide which of the puppets made a mistake. Thereby the focus of the elicited response was changed from detecting errors in general to the more specific determination of *where* in the puppets' play the error had occurred.

### Method

A different sample of forty-eight 4-year-olds (48–59 months, mean age  = 54 months, 24 boys) was recruited from the same local database as in Study 1 (11 additional children were excluded from the final sample, three due to uncooperative behavior, four for not passing the training phase, four due to experimenter errors or technical failure). Children were native German speakers, came from diverse socioeconomic backgrounds and were tested either in their daycare centers or in the child lab facilities.

### Design & Procedure

As in Study 1, the rationale for the *warm-up phase* was to familiarize children with the fact that there might be verbal and/or action mistakes on the parts of the puppets. The only modification compared to Study 1 shifted the focus from the detection of puppets' errors in general towards a specific differentiation between correct and incorrect actions of the two puppets. Hence, the exact same warm-up games as in Study 1 were used but with the difference that in Study 2 the puppets always played together performing comparable actions. In case of the picture book, the puppets both claimed that they knew the book already, and therefore each stated its opinion on what picture would appear on the next page, in advance. That means the puppets played two rounds, both times making divergent utterances about which picture would show up, and with each puppet predicting the outcome correctly once. In the game with hammer and balls, the puppets performed simultaneously with duplicate apparatuses. Again, this game was played for two rounds with one puppet pushing the correct ball while the other simultaneously made a mistake by hammering on the wrong color (roles were alternated).

To establish the forced choice paradigm, after each round E1 presented two cards to the child, each depicting one of the puppets together with a speech bubble (or with a hammer in hand, respectively (for details regarding the material, see Materials S3). E1 then explained: “Look at these cards. This is the sheep saying something (*or*: the sheep hammering) and here is the hedgehog saying something (*or*: the hedgehog hammering). Show me what was wrong!” Dependent on the child's readiness to point to the correct card, E1 used up to three additional prompts in order to encourage the child to respond in form of pointing to one of the cards. Four children failed to report on the mistake by pointing to the cards, and were therefore excluded from the final sample.

Design and procedure of the *test game* were identical to Study 1, except for two changes: first, the object-container pairs were changed into sets of two-dimensional sorting games in order to facilitate children's handling of containers. This afforded children to choose one out of only two possible containers (e.g. the puzzle with round holes for marbles or the one where cubic objects fit in). Second, after the puppets performance in each trial E1 presented two cards to the child, similarly to the warm-up phase; the speech act-card showed the respective puppet with a speech bubble, the action-card showed the actor puppet manipulating the slide. The cards were placed in front of the corresponding room of the puppet house, i.e. if, say, the sheep was the speaker and in the left room, then the sheep's speech-act card on the left, and the hedgehog's action card on the right from the child's point of view. As children were used to the pointing task from the warm-up already, E1 only looked at the cards (alternating her gaze between the pictures) asking “Show me what was wrong!”. Or, in case of a child responding verbally however, she insisted “Just *show* me what was wrong!”. As in Study 1, children received a total of eight trials, four *future-assertive (prediction)* and four *future-directive (imperative)* trials which were presented in successive blocks. The order of blocks, the assignment of games to conditions, and the order of the puppet's roles within each block (speaker vs. actor, alternating over trials) were counterbalanced across children.

### Coding

Children's responses were coded as pointing to the speech act-card or to the action-card (or as behavior that did not fall in either of these categories if children did not point at all, or failed to follow the forced choice in some other way, which was very rare). In case a child's first response was not a clear pointing gesture (e.g. moving the hand over both cards while pointing) or in case of a child switching from her first choice to the other card, E1 repeated her request up to two times until the child produced a clear response which was then coded as the child's final and valid decision.

The directed hypotheses and rationale for the statistical analyses were the same as in Study 1.

### Results and Discussion

**Figure 3:**
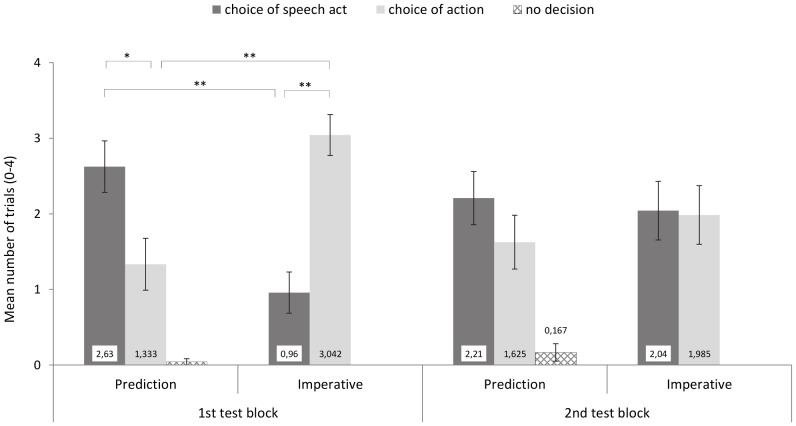
Responses to mismatches in Study 2 (forced-choice measure). ean sum scores of trials (0–4) with each type of decision (choice of picture depicting speech act, action, or no decision) as a function of condition (Prediction, Imperative) and order of test blocks (conditions were presented in blocks, with the order counterbalanced).

For each child, for each of the decisions (speech act-card/action-card) and the non-decisions, sum scores across the 4 trials per condition were computed. The means of these sum scores as a function of condition and order of test blocks are depicted in Fig. 3.

Preliminary analyses suggested clear order effects: 2 (condition; within subjects) x 2 (order of test blocks; between subjects) mixed-factors ANOVAs on the mean number of trials with actor-card-decisions and speaker-card-decisions, respectively, yielded a significant condition X order of test block interaction effect in the case of actor-card-decisions, *F*(1, 46) = 18.94, *p*<.001, *η_p_* ^2^ = .29, and speaker-card-decisions, *F*(1, 46) = 15.68. *p*<.001, *η_p_* ^2^ = .25. Children's performance in the second block was significantly influenced by their behavior in the first block and was thus difficult to interpret. Why children showed this order effect we cannot tell from the present data. One possibility is that it was simply due to response perseveration: Given that the present tasks pose quite some performance demands, for example on working memory (keeping track of who said what when, and who did what when), children might have been overwhelmed after a while and simply stuck to previously successful answers (always pointing to the speaker-card or always to the actor-card). Alternatively, children might have suffered from more cognitive perseveration: after some trials in which there were always speech act mistakes, for example, they might have found it difficult to disengage from thinking of the speech act – action mis-matches as due to mistakes on the part of the speaker. Subsequently, in the fashion of Piagetian assimilation, they then over-generalized their mini-theory that truthfully captured the first trials (e.g. “speakers are always wrong here”) inappropriately to trials in the second block. Clearly, future research is needed to explore these possibilities.

Regarding subsequent data processing, the focus for statistical analyses was on the more valid data of first test block (now in a between-subjects design such that half of the subjects was tested in the imperative condition and the other half in the prediction condition). First, analyses of children's choice of card within each condition revealed a significantly higher rate of speech-act than action card choices in the prediction condition, *t*(23) = −1.89, *p*<.05, *d* = .77, and the reverse pattern in the imperative condition, *t*(23) = 3.82, *p*<.001, *d* = 1.56. These results were confirmed by non-parametric analyses for the prediction condition (Wilcoxon test, *T* = 178, *p*<.05, *r* = .36), and for the imperative condition (Wilcoxon test, *T* = 38, *p*<.01, *r* = .61). Second, analyses of each type of choice as a function of condition revealed that the action-card was chosen more often by children in the imperative condition than by children in prediction condition, *t*(46) = 3.89, *p*<.001, *d* = 1.15. Analogously, the speech act-card was chosen more often in the prediction condition than in the imperative condition, *t*(46) = 3.89, *p*<.001, *d* = 1.13. Again, non-parametric tests confirmed these results for speech-act card choices in the imperative and prediction condition (Mann Whitney U-test, *U* = 440.5, *z* = 3.28, *p*<.001, *r* = .47) and for action-card choices, respectively (Mann Whitney U-test, *U* = 133.5, *z* = 3.33, *p*<.001, *r* = .48).

In sum, then, the modified response measure introduced in Study 2 succeeded in eliminating the high rate of ambiguous responses reported in Study 1: the clear results for direction-of-fit recognition in situations of mismatches between imperative speech-acts and actions were replicated in Study 2. And now the rather unclear pattern of responses in the prediction conditions found in Study 1 turned into a distinct preference to recognize the speech-act as source of the mismatch in future-assertive trials of Study 2.

## General Discussion

The present findings show that by 4 years of age, children have developed a basic understanding of the underlying normative structure of future-directed speech acts with opposing direction of fit. They differentially track mismatches between the content of speech-acts and temporally successive events in the world and are ready to intervene appropriately: In case of imperatives, the majority of children verbally criticized (Study 1) and pointed to (Study 2) the actor for being responsible for the mismatch. In the case of predictions, they criticized the speaker more often than they did after unfulfilled imperatives (Study 1); and they explicitly identified the speaker as the source of the mistake under conditions of suitable prompting (Study 2). In sum, the present results demonstrate that children understand that thoughts and speech acts have specific normative outreach into the future as a function of their direction-of-fit. And they demonstrate this at a much earlier age than suggested by previous research on speech act development. Probably this difference in findings between the present and previous studies is partly due to the very different methodologies: in contrast to the verbal interviews based on complex narratives in earlier work, the present studies used a much simpler action-based methodology. Another reason might be that the contrast pair between other-directed speech acts used here (predictions versus imperatives) might be inherently easier to grasp than the contrast between first person future-directed speech acts (predictions versus promises) that has mostly been studied in previous work (see below).

The present findings add to previous research in several ways: regarding children's grasp of normativity, they add to our knowledge that children understand the logical difference between different *synchronic* directions of fit of different speech acts by showing that children understand the *diachronic* normative structure of direction of fit over time. Regarding temporal cognition, they add to our knowledge of the development of thinking about matters in time by revealing the normative side of early future-oriented thought. Children understood that thought and speech can reach out into the future in normative ways: actions at one time can normatively bind and commit agents over time.

The present findings thus reveal reasoning about temporal-normative relations in young children. What cognitive representations and processes exactly underlie such reasoning is an interesting question for future research. This is structurally parallel to other domains of temporal cognition. For example, in the recent literature on mental time travel in humans and other animals, it has been intensively debated which types of tasks require which levels of (implicit or explicit) representation of time, e.g., [5], [35], [36]. Everyone agrees, for instance, that episodic memory involves representations of one's own past. But there has been considerable disagreement about the type of representation it requires: some think it requires explicit representations both of one's own past events as past events and of the way they causally relate to one's present memories [37], [38]. Others, in contrast, have argued that episodic memory is well possible without such sophisticated representational machinery. Instead, it need only represent explicity where, when and what happened while only implicitly representing the relation of these events to the present, e.g., [35], [39]. In a similar vein, the present findings minimally show that young children represent temporal-normative relations over time. But they leave open for future studies the question what exactly is the nature, format and content of these representations.

Relatedly, it remains to be explored in future studies how sophisticated and flexible the tracking of trans-temporal normative relations as documented here is. First, the distance that the speech acts reached out into the future in the present studies was in fact small, as the speech acts referred to the rather immediate future. This is in contrast to much research on mental time travel and temporal cognition, in which planning for and mentally traveling to the more distant future is investigated. It thus remains to be clarified whether similar cognitive foundations underlie these different forms of thinking about the future differing in the temporal distance between present and future. Second, the studies here suggest that children track the normative relations between one person's speech act at time 1 and another person's actions at time 2. An obvious question regards the relations within one person between her words today and her deeds tomorrow. When do children develop an understanding of the analogous difference in normative structure between first-person predictions (“When the wind comes, I will fall off my bike”) and promises/declarations of intention (“When the summer comes, I will cut my hair”)? This, it should be noted, is very difficult to study in an equally stringent way for practical reasons: It is very difficult to find plausible scenarios where the very same propositional content “I will X” can be used to declare an intention and make a prediction. Typically, “I will” is used to declare an intention when X is a verb for an intentional action (“cut one's hair”) and is used to merely make a prediction when X is a verb for a mere happening (“fall off one's bike”).

A broader open question is how children come to represent intentional normative self-binding over time in its complex subtleties. Our intentional states and speech acts today do have normative implications for actions tomorrow and do bind us and others over time – but they do not do so in inflexible and slavish ways, e.g., [40]. We can change our minds. Correspondingly, it is one thing not to live up to one's own or others' standards set yesterday by failing to fulfill one's past future-directed intention or another's reasonable request. It is quite another thing, though, to give up an intention one had or to decide not to comply with a request. In all of these cases there are mismatches between mental states/speech acts at time 1 and actions at time 2 – but only in the former cases are there any mistakes involved.

## Supporting Information

Materials S1 **Examples of object-container pairs (Study 1).**
(TIF)

Materials S2 **Sorting games (Study 1) and slide (Study 1, Study 2).**
(TIF)

Materials S3 **Cards used in the forced-choice paradigm (Study 2).** Upper row: cards used to introduce the forced choice paradigm in the warm-up phase, row below: cards used in test trials of Study 2.
(TIF)

Table S1 **Script (Study 1, Study 2).** Script of the puppet play preceding the response phases in prediction and imperative conditions.
(TIF)
