# Supplementary figures and images for: Young Children Understand the Normative Implications of Future-Directed Speech Acts
Source: PLoS One. 2014 Jan 29;9(1):e86958. doi: 10.1371/journal.pone.0086958 (PMC3906105; doi:10.1371/journal.pone.0086958)

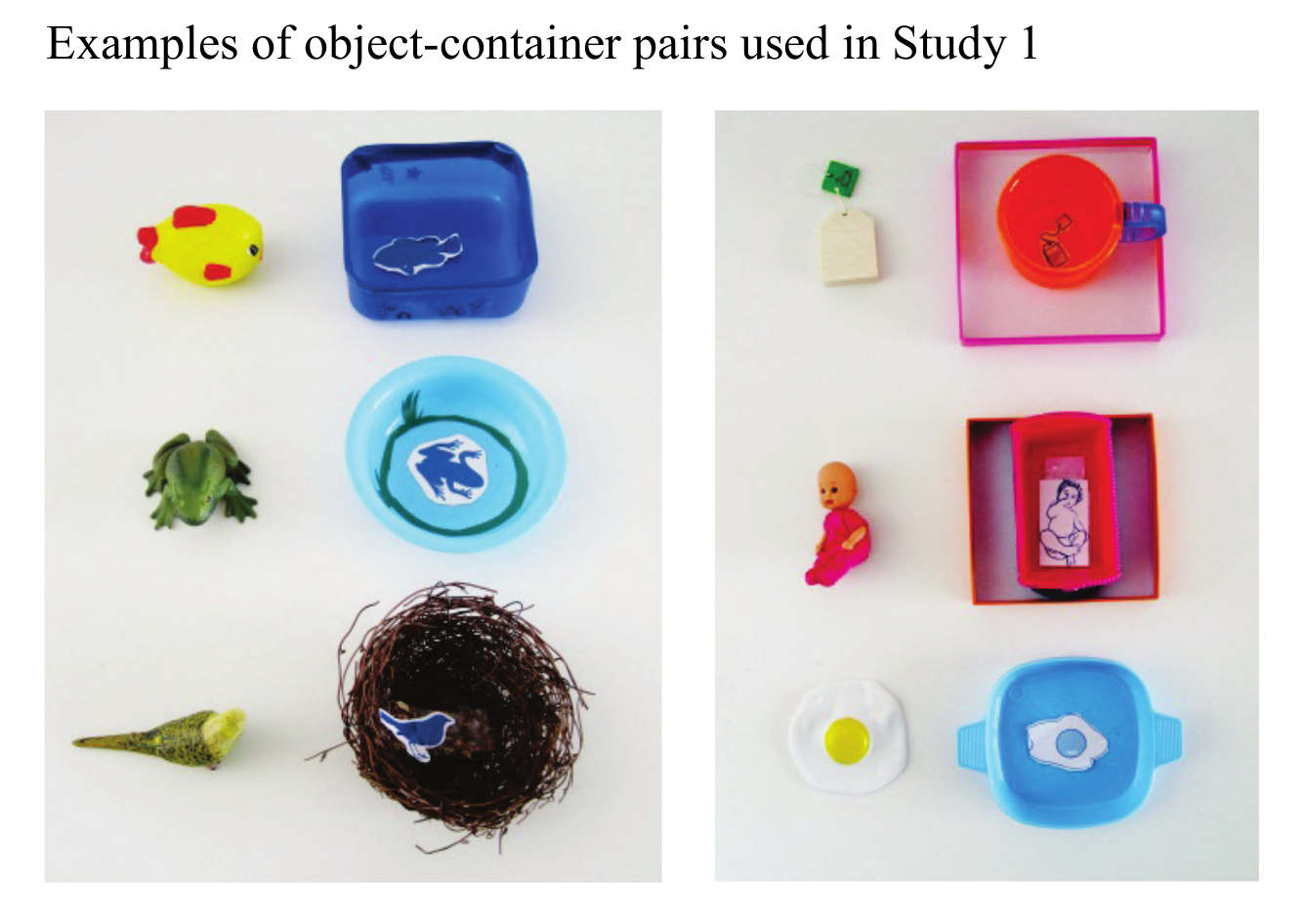

Supplement: Materials S1 — Examples of object-container pairs (Study 1). (TIF) [file pone.0086958.s001.tif]

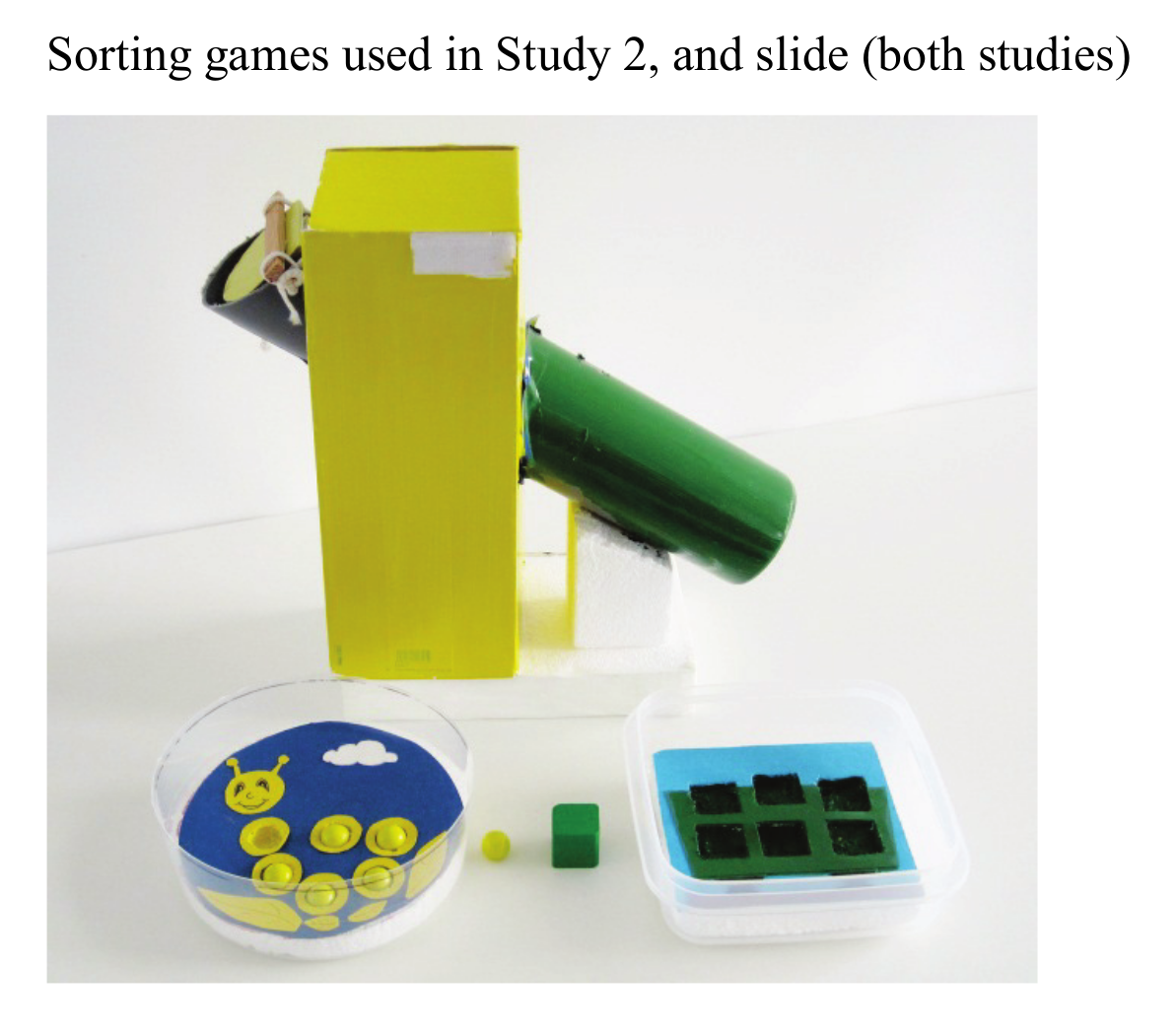

Supplement: Materials S2 — Sorting games (Study 1) and slide (Study 1, Study 2). (TIF) [file pone.0086958.s002.tif]

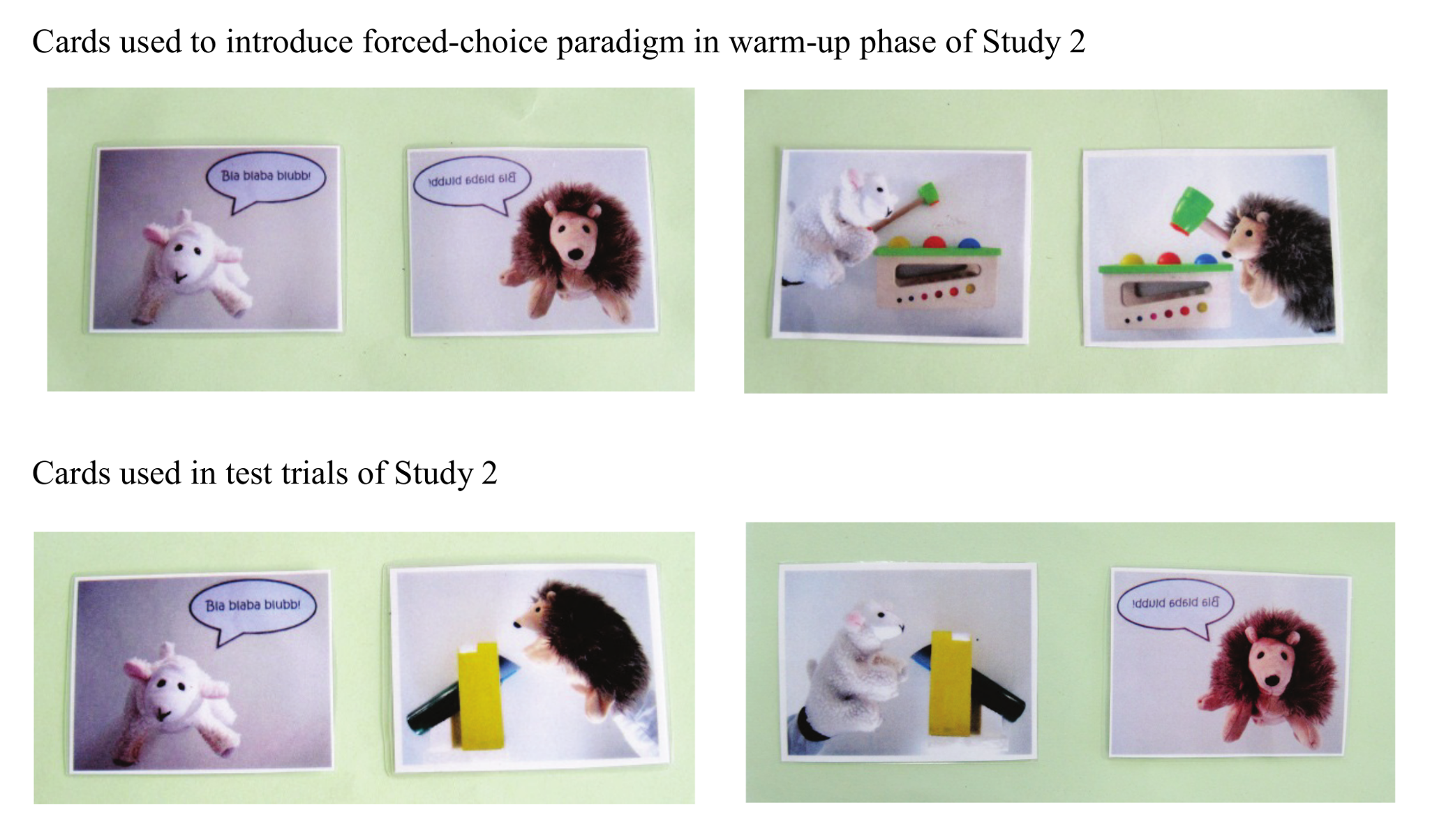

Supplement: Materials S3 — Cards used in the forced-choice paradigm (Study 2). Upper row: cards used to introduce the forced choice paradigm in the warm-up phase, row below: cards used in test trials of Study 2. (TIF) [file pone.0086958.s003.tif]

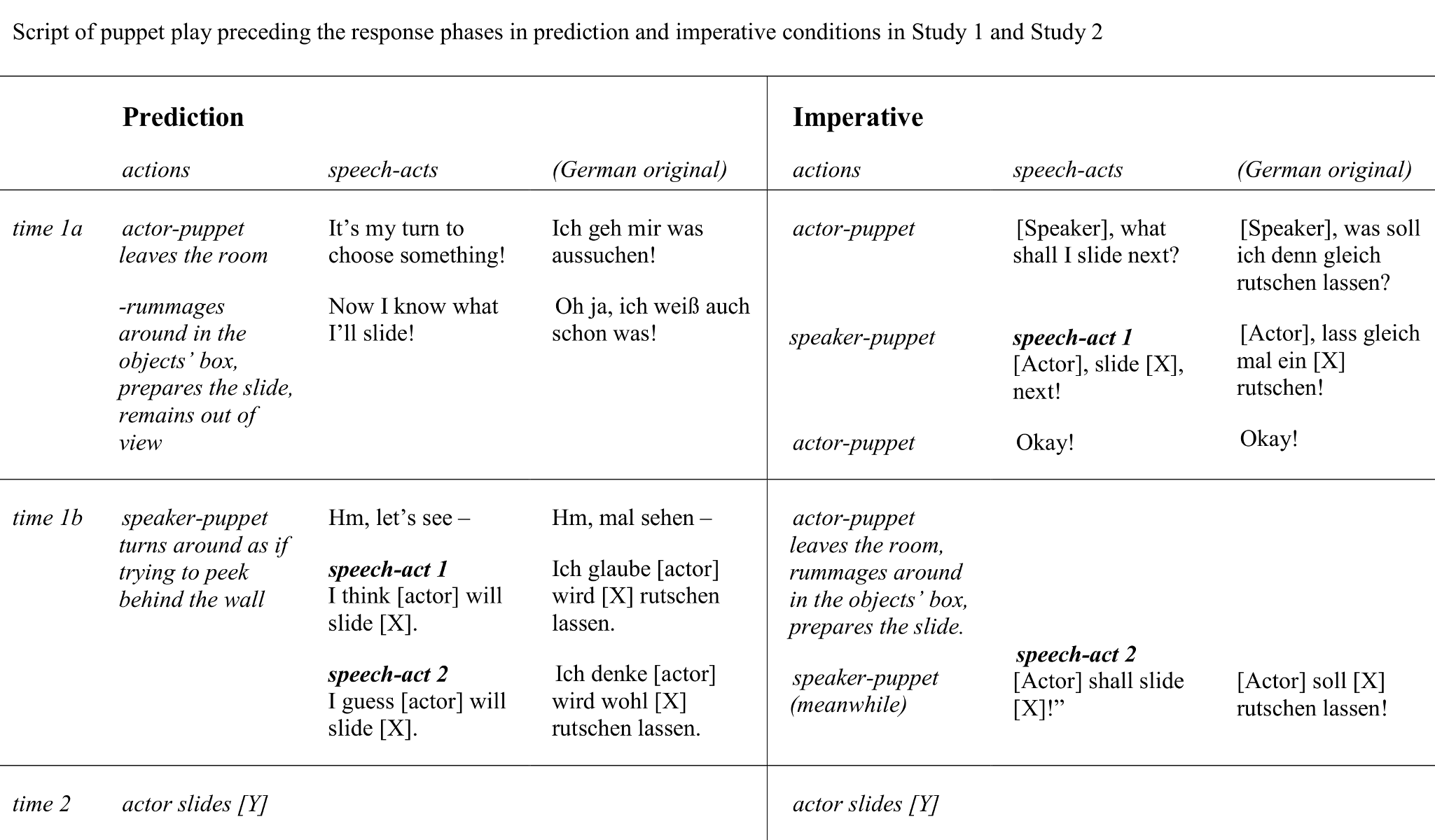

Supplement: Table S1 — Script (Study 1, Study 2). Script of the puppet play preceding the response phases in prediction and imperative conditions. (TIF) [file pone.0086958.s004.tif]
